# Incident dementia risk among patients with type 2 diabetes receiving metformin versus alternative oral glucose-lowering therapy: an observational cohort study using UK primary healthcare records

**DOI:** 10.1136/bmjdrc-2023-003548

**Published:** 2024-01-25

**Authors:** William Doran, Louis Tunnicliffe, Rutendo Muzambi, Christopher T Rentsch, Krishnan Bhaskaran, Liam Smeeth, Carol Brayne, Dylan M Williams, Nish Chaturvedi, Sophie V Eastwood, Susanna J Dunachie, Rohini Mathur, Charlotte Warren-Gash

**Affiliations:** 1 Faculty of Epidemiology and Population Health, London School of Hygiene & Tropical Medicine, London, UK; 2 Department of Internal Medicine, Yale School of Medicine, New Haven, Connecticut, USA; 3 Cambridge Public Health, University of Cambridge, Cambridge, UK; 4 MRC Unit for Lifelong Health and Ageing, University College London, London, UK; 5 NDM Centre for Global Health Research, Nuffield Department of Medicine, University of Oxford, Oxford, UK; 6 NIHR Oxford Biomedical Research Centre, Oxford University Hospitals NHS Foundation Trust, Oxford, UK; 7 Centre for Primary Care, Wolfson Institute of Population Health, Queen Mary University of London, London, UK

**Keywords:** Dementia, Diabetes Mellitus, Type 2, Metformin, Electronic Health Records

## Abstract

**Introduction:**

4.2 million individuals in the UK have type 2 diabetes, a known risk factor for dementia and mild cognitive impairment (MCI). Diabetes treatment may modify this association, but existing evidence is conflicting. We therefore aimed to assess the association between metformin therapy and risk of incident all-cause dementia or MCI compared with other oral glucose-lowering therapies (GLTs).

**Research design and methods:**

We conducted an observational cohort study using the Clinical Practice Research Datalink among UK adults diagnosed with diabetes at ≥40 years between 1990 and 2019. We used an active comparator new user design to compare risks of dementia and MCI among individuals initially prescribed metformin versus an alternative oral GLT using Cox proportional hazards regression controlling for sociodemographic, lifestyle and clinical confounders. We assessed for interaction by age and sex. Sensitivity analyses included an as-treated analysis to mitigate potential exposure misclassification.

**Results:**

We included 211 396 individuals (median age 63 years; 42.8% female), of whom 179 333 (84.8%) initiated on metformin therapy. Over median follow-up of 5.4 years, metformin use was associated with a lower risk of dementia (adjusted HR (aHR) 0.86 (95% CI 0.79 to 0.94)) and MCI (aHR 0.92 (95% CI 0.86 to 0.99)). Metformin users aged under 80 years had a lower dementia risk (aHR 0.77 (95% CI 0.68 to 0.85)), which was not observed for those aged ≥80 years (aHR 0.95 (95% CI 0.87 to 1.05)). There was no interaction with sex. The as-treated analysis showed a reduced effect size compared with the main analysis (aHR 0.90 (95% CI 0.83 to 0.98)).

**Conclusions:**

Metformin use was associated with lower risks of incident dementia and MCI compared with alternative GLT among UK adults with diabetes. While our findings are consistent with a neuroprotective effect of metformin against dementia, further research is needed to reduce risks of confounding by indication and assess causality.

**WHAT IS ALREADY KNOWN ON THIS TOPIC**

- Diabetes is a risk factor for dementia in later life with an estimated population attributable fraction of 1%.
- Evidence that metformin therapy reduces the risk of dementia in diabetes is disputed.

**WHAT THIS STUDY ADDS**

- We found that metformin users experienced a lower risk of incident all-cause dementia and mild cognitive impairment than users of other oral glucose-lowering therapies (GLTs) in a large, unselected UK primary care population.
- This risk reduction was seen only in individuals aged under 80 years, which is consistent with previous studies. Our findings were robust across a broad range of sensitivity analyses, but risk reduction attenuated when we applied additional control for exposure misclassification.

**HOW THIS STUDY MIGHT AFFECT RESEARCH, PRACTICE OR POLICY**

- This study highlights a need for further research including randomized controlled trials and instrumental variable analyses to strengthen causal inference.
- The development of dementia prevention strategies is a key concern for current policymakers: optimizing GLT for individuals with diabetes may help to improve brain health.

## Introduction

4.2 million individuals are living in the UK with type 2 diabetes mellitus (hereafter referred to as ‘diabetes’) and prevalence is increasing due to underlying trends in physical inactivity, obesity and population aging.^1^ Diabetes is an established dementia risk factor through mechanisms including insulin resistance, chronic neuroinflammation and microvascular dysfunction.^2 3^ Globally, dementia already costs US$1 trillion annually and the number of individuals living with dementia in the UK is expected to increase from 850 000 individuals to 1.2 million by 2040.^2^ Low and middle-income countries are reporting rapid increases in dementia incidence consistent with their demographic transition, but high-income countries such as the UK already have a high burden of dementia and multimorbidity in line with their older population structures.^2^ There is an urgent need for dementia prevention strategies, and considering risk factor modification of diabetes is highly relevant to all socioeconomic settings.

Individuals with diabetes are 1.5–2 times more likely to be diagnosed with a cognitive disorder including mild cognitive impairment (MCI) or dementia.^3^ Dementia risk appears to increase with duration of diabetes and poor glycemic control, but there is inconsistent evidence on whether this is modifiable by glucose-lowering therapy (GLT) such as metformin. Metformin, a biguanide in widespread global use, has been recommended as first-line treatment for diabetes by the National Institute for Health and Care Excellence (NICE) since 2002.^4 5^ Prior to this, alternative GLTs such as sulfonylureas were frequently used as first-line treatment. Traditionally, metformin’s principal mechanisms were understood to be suppression of hepatic gluconeogenesis and enhancement of peripheral insulin sensitivity via AMPK activation.^6^ However, it also exhibits pleiotropic effects including modifying the gut microbiome, immune function and inflammatory mechanisms, which could plausibly modify the pathogenesis of dementia in diabetes.^7 8^

The association between metformin therapy and incident dementia in patients with diabetes is contested: two recent meta-analyses found evidence that metformin therapy was associated with lower incident dementia risk, but a third reported a pooled null effect.^9–11^ Many of the included studies were prone to bias due to the use of cross-sectional designs and lack of active comparators. Subsequent to these meta-analyses, Newby *et al* estimated a 20% lower incident dementia risk associated with metformin use using an active comparator new user design in US health records.^12^ Here, we aimed to investigate the association between metformin therapy and incident all-cause dementia and MCI using primary care records from the UK Clinical Practice Research Datalink (CPRD).^13^

## RESEARCH DESIGN AND METHODS

CPRD Gold includes longitudinal primary care records from practices using Vision software covering a large, unselected study population, which is demographically representative of the UK.^13^ It contains details of symptoms, coded diagnoses, test results, referrals and drug prescriptions. It also includes well-established linkages to Office for National Statistics mortality data, Index of Multiple Deprivation (IMD) and Hospital Episode Statistics (HES) Admitted Patient Care data.^13^

We compared new users of metformin with new users of alternative GLTs in an active comparator new user (ACNU) design. This aims to emulate the advantages of randomized controlled trials in observational settings and mitigates potential confounding by indication and healthy user bias. It excludes individuals without an indication for treatment or with important contraindications including frailty. It also ensures that participants are aligned at a common time point (initiation of treatment).^14^

### Source population and cohort identification

**Figure 1:**
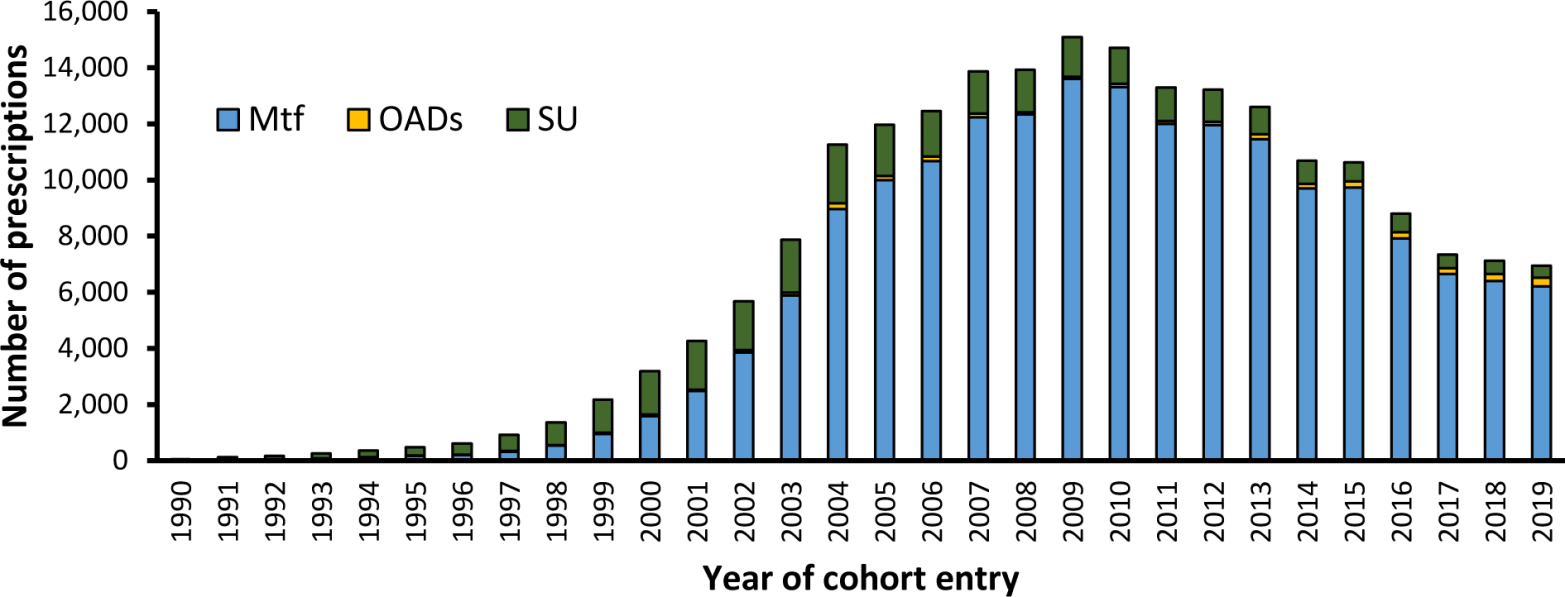
Distribution of first prescriptions of oral GLT over time. NICE guidance changed in 2002 to recommend metformin as first-line treatment for diabetes. GLT, glucose-lowering therapy; Mtf, metformin; NICE, National Institute for Health and Care Excellence; OADs, other anti-diabetic drugs; SU, sulfonylurea.

The study population included adults who were registered at a CPRD-eligible general practice between January 1, 1990 and December 31, 2019 with a new diagnosis of diabetes at ≥40 years old recorded at least 12 months after registration. Eligible individuals required a record of GLT with first prescription on or at any time after the diabetes diagnosis date and no history of dementia or MCI at first prescription (index date) (online supplemental methods and figure 1).

10.1136/bmjdrc-2023-003548.supp1 Supplementary data

### Follow-up

The index date for follow-up was the first prescription date for GLT. For the dementia outcome, follow-up continued until an instance of death, dementia, CPRD de-registration or December 31, 2019, whichever was first. For MCI, follow-up continued until an instance of death, dementia, MCI, CPRD de-registration or December 31, 2019, whichever was first; this ensured non-sensical diagnoses of MCI made after diagnoses of dementia were discounted. Follow-up was restricted to the end of 2019 to exclude unknown impacts of the SARS-CoV-2 pandemic.

### Exposure and outcome

The exposure was defined as the first CPRD-recorded prescription of metformin or alternative GLT among individuals with no prior record of either. Eligible GLT included any formulation of: metformin, alpha-glucosidase inhibitors, sulfonylureas, dipeptidyl peptidase 4 inhibitors, glucagon-like peptide-1 analogs, thiazolidinediones, sodium-glucose cotransporter 2 (SGLT-2) inhibitors and metaglinides. We applied a ‘first treatment carried forward’ approach, meaning that individuals who first began metformin or alternative GLT remained in their original exposure group regardless of treatment non-adherence, intentional cessation or commencement of any additional GLT (we found no instances of metformin therapy and alternative GLT being initiated simultaneously).

All-cause dementia (primary outcome) and MCI (secondary outcome) were defined using extensive, previously established Read code lists based on clinical diagnoses (dementia) or diagnoses and symptoms (MCI) recorded in primary care. All-cause dementia included all known subtypes such as Alzheimer’s disease, vascular dementia, Lewy body dementia as well as undifferentiated dementia. International Classification of Diseases Tenth Revision (ICD-10) codes from hospital records were additionally used to identify dementia outcomes in a sensitivity analysis restricted to individuals with linked data only. In this analysis, the dementia date was taken from the earliest record in either CPRD or HES.

### Covariates

Using year of birth, we assumed a July 2 birth date for all participants. Individual-level and general practitioner (GP)-level IMDs were defined as quintiles obtained from Office for National Statistics linkage. Self-reported ethnicity was classified into five categories: white, South Asian, black, mixed or other and unknown. Smoking status was classified as current, former, and never. Body mass index was taken from the measurement closest to the index date in the 12 months prior or 3 months after and was categorized according to the WHO cut-offs. ‘Baseline’ hemoglobin A1c (HbA1c) in International Federation of Clinical Chemistry units was measured within 6 months prior to the index date and classified into broad 20 mmol/mol categories.

21 additional covariates were collected that describe participants’ health status prior to the index date and reflect known or hypothesized risk factors for dementia. These included: statin use, antihypertensive use, hypertension, asthma, chronic obstructive pulmonary disease, liver disease, coronary heart disease, peripheral vascular disease, stroke, diabetic retinopathy, neuropathy, brain injury, depression, autoimmune disease, chronic kidney disease, heart failure, alcohol excess, skin and soft tissue infection, urinary tract infection, lower respiratory tract infection and sepsis. Code lists are available on London School of Hygiene & Tropical Medicine Data Compass: https://datacompass.lshtm.ac.uk/id/eprint/3402/.

### Statistical analysis

Analysis was completed using Stata/SE V.17 (Statacorp). Baseline characteristics were described overall and by exposure status with frequency counts and percentages apart from age, which was described with the median value and IQR. We hypothesised that missing data were likely missing not at random, meaning that multiple imputation was unsuitable.^15^ There were 115 092 (54.4%) missing entries for individual-level IMD, which were replaced with GP-level IMD. Three other covariates had high proportions of missingness: ethnicity (50.9%), baseline HbA1c (16.9%) and smoking status (13.8%). Crude rates of incident dementia and MCI were calculated overall and by age and calendar time periods with 95% CIs estimated according to the Poisson distribution.

We conducted a single-failure survival analysis using Cox regression with fixed effects and age as the underlying time scale for dementia and MCI. Age-adjusted, minimally adjusted (adjusted for age as time scale plus sex and calendar time) and fully adjusted models (all covariates) were fitted. The fully adjusted models were fitted using a backwards approach, in which all baseline covariates were considered as potential confounders, informed by our directed acyclic graph (online supplemental figure 2). Covariates with >20% missingness (ethnicity) were excluded. Lexis expansion was used to generate 5-year calendar time bands, which were used to control for secular effects. The three earliest time bands were merged after encountering event sparsity. We calculated root mean-squared error (RMSE) to indicate multicollinearity and bias. If this increased between the minimally adjusted and fully adjusted model for the primary outcome, covariates were individually dropped from repeated regressions to identify which could be excluded to achieve the largest reduction in RMSE. This model reduction process was repeated until the RMSE of the fully adjusted model was lower than that of the minimally adjusted model. The regression models for MCI were specified with the same covariates as for dementia to ensure that estimates were comparable. The Schoenfeld residuals test and log-log Kaplan-Meier survival plots were used to evaluate evidence for non-proportional hazards.

We decided a priori to assess for potential interaction by age and sex as there is plausible evidence to support this.^16 17^ We specified stratified models and used the likelihood ratio test (LRT) to assess evidence for interaction. We undertook multiple sensitivity analyses to assess the robustness of our findings. To minimize misclassification of pre-existing dementia as incident dementia, we restricted follow-up to exclude dementia diagnoses up to 2 years after the index date. Then, we performed subgroup analysis for individuals started on treatment ≥2004 and ≥2012. CPRD data quality meaningfully improved after the advent of the NICE Quality of Outcomes Framework (QOF) in 2004^18^ and the first SGLT-2 inhibitor (dapagliflozin) was approved for UK use in 2012. We restricted our analysis to individuals with HES linkage as we hypothesized they may have improved data quality for covariates and dementia.^19^ After this, we performed a post-hoc ‘as-treated’ analysis where participants were censored 30 days (a typical medication supply) after their last prescription of metformin or non-metformin GLT. Finally, we applied serial restrictions on calendar period to assess whether spurious calendar effects were biasing effect estimates.

## Results

There were 211 396 eligible individuals with a median follow-up of 5.4 years. Of these, 179 333 (84.8%) initiated treatment with metformin and had a median follow-up of 5.2 years, while 32 063 (15.2%) initiated alternative GLT and had a median follow-up of 6.9 years. The proportion of metformin initiators increased steadily over the study period from 30.0% of participants between 1990 and 1994 to 90.4% of participants by 2015–2019. 90.3% of those initiating an alternative GLT were prescribed a sulfonylurea (figure 1). We noted that 51.2% of individuals initiated on metformin were later prescribed an alternative GLT and 69.0% of individuals initiated on alternative GLT were later prescribed metformin.

### Characteristics of baseline population

The cohort was majority male (57.2%), with a median age of 63 years at baseline (IQR: 54–71 years). Metformin users were younger (median age 62 years vs 66 years), more likely to be overweight/obese and to be prescribed statins (65.8% vs 48.4%) or antihypertensives (69.1% vs 65.2%) than those on alternative GLTs. They also had lower baseline HbA1c measurements (median 63.9 mmol/mol, IQR 55.2–80.3) compared with alternative GLT users (median HbA1c 72.7 mmol/mol, IQR 58.5–96.7). Alternative GLT users were more likely to have a history of coronary heart disease, peripheral vascular disease and heart failure (table 1).

**Table 1:** Baseline characteristics of cohort overall and by GLT type

Variable		Overall N (%_n_)	Metformin N (%_n_)	Other GLTs N (%_n_)
Total		211 396 (100.0)	179 333 (84.8)	32 063 (15.2)
Age at entry	Median (IQR)	63 (54–71)	62 (54–71)	66 (56–75)
Sex	Male	120 902 (57.2)	102 127 (56.9)	18 775 (58.6)
	Female	90 494 (42.8)	77 206 (43.1)	13 288 (41.4)
Ethnicity	White	94 470 (91.0)	81 707 (91.0)	12 763 (91.1)
	South Asian	5352 (5.2)	4677 (5.2)	675 (4.8)
	Black	2253 (2.2)	1921 (2.1)	332 (2.4)
	Mixed or other	1744 (1.7)	1511 (1.7)	233 (1.7)
	Missing	107 577 (50.9*)	89 517 (49.9*)	18 060 (56.3*)
Index of Multiple Deprivation	1 (poorest)	35 512 (16.8)	29 933 (16.7)	5579 (17.4)
	2	37 817 (17.9)	31 624 (17.6)	6193 (19.3)
	3	43 983 (20.8)	37 318 (20.8)	6665 (20.8)
	4	45 679 (21.6)	39 124 (21.8)	6555 (20.4)
	5 (wealthiest)	48 405 (22.9)	41 334 (23.0)	7071 (22.1)
Baseline HbA1c (mmol/mol)	<48	13 120 (7.5)	11 353 (7.3)	1767 (8.3)
	48–67.9	86 038 (49.0)	78 682 (50.9)	7356 (34.7)
	68–87.9	42 042 (23.9)	36 852 (23.9)	5190 (24.5)
	88–107.9	21 153 (12.0)	17 591 (11.4)	3562 (16.8)
	>108	13 374 (7.6)	10 036 (6.5)	3338 (15.7)
	Missing	35 669 (16.9*)	24 819 (13.8*)	10 850 (33.8*)
Body mass index (BMI) (kg/m^2^)†	Underweight	566 (0.3)	237 (0.1)	329 (1.2)
	Normal weight	19 226 (10.2)	12 334 (7.6)	6892 (26.1)
	Overweight	58 922 (31.3)	48 770 (30.1)	10 152 (38.5)
	Obesity class I	58 380 (31.0)	52 832 (32.6)	5548 (21.0)
	Obesity class II, III	51 206 (27.2)	47 756 (29.5)	3450 (13.1)
	Missing	23 096 (10.9*)	17 404 (9.7*)	5692 (17.8*)
Smoking status	Never	80 894 (44.3)	70 059 (44.3)	10 835 (44.9)
	Current	33 907 (18.6)	29 223 (18.5)	4684 (19.4)
	Ex	67 517 (37.0)	58 894 (37.2)	8623 (35.7)
	Missing	29 078 (13.8*)	21 157 (11.8*)	7921 (24.7*)
Alcohol misuse		4143 (2.0)	3522 (2.0)	621 (1.9)
Statin		133 510 (63.2)	117 997 (65.8)	15 513 (48.4)
Antihypertensive		144 892 (68.5)	123 982 (69.1)	20 910 (65.2)
Hypertension		107 618 (50.9)	92 783 (51.7)	14 835 (46.3)
Asthma		33 733 (16.0)	29 250 (16.3)	4483 (14.0)
COPD		23 362 (11.1)	19 466 (10.9)	3896 (12.2)
Liver disease		5032 (2.4)	4296 (2.4)	736 (2.3)
Coronary heart disease		34 187 (16.2)	27 841 (15.5)	6346 (19.8)
Peripheral vascular disease		4322 (2.0)	3430 (1.9)	892 (2.8)
Stroke		768 (0.4)	628 (0.4)	140 (0.4)
Diabetic retinopathy		12 797 (6.1)	11 098 (6.2)	1699 (5.3)
Neuropathy		4560 (2.2)	3866 (2.2)	694 (2.2)
Brain injury		2044 (1.0)	1780 (1.0)	264 (0.8)
Depression		56 270 (26.6)	49 195 (27.4)	7075 (22.1)
Autoimmune disease		16 871 (8.0)	13 498 (7.5)	3373 (10.5)
Chronic kidney disease		894 (0.4)	332 (0.2)	562 (1.8)
Heart failure		17 806 (8.4)	13 526 (7.5)	4280 (13.3)
Skin and soft tissue infection		42 387 (20.1)	36 845 (20.5)	5542 (17.3)
Urinary tract infection		36 574 (17.3)	31 081 (17.3)	5493 (17.1)
Lower respiratory tract infection		83 315 (39.4)	71 633 (39.9)	11 682 (36.4)
Sepsis		1964 (0.9)	1550 (0.9)	414 (1.3)

%n refers to the % among those without missing data

*% of total.

†BMI category definitions (kg/m^2^): underweight <18.5, normal weight 18.5–24.9, overweight 25–29.9, obesity class I 30–34.9, obesity class II and III ≥35.

COPD, chronic obstructive pulmonary disease; GLT, glucose-lowering therapy; HbA1c, hemoglobin A1c; %_n_, % of non-missing.

### Description of the outcomes

We observed 6642 diagnoses of dementia and 10 804 diagnoses of MCI during follow-up. The overall crude incidence rates were 5.0 per 1000 person-years for dementia and 7.6 per 1000 person-years for MCI. Incidence rates of dementia and MCI increased with age: the dementia incidence rate was 0.05 (95% CI 0.02 to 0.11) per 1000 person-years among individuals 40–49 years old, but 34.18 (95% CI 31.36 to 37.25) per 1000 person-years in those ≥90 years old. Dementia and MCI incidence rates also increased across successive calendar periods. Dementia incidence was 1.20 (95% CI 1.01 to 1.43) per 1000 person-years in 1990–2004, but 7.05 (95% CI 6.79 to 7.31) between 2015 and 2019. This pattern appeared preserved when conditioning on age (online supplemental table 1).

### Association of new users of metformin versus alternatives with all-cause dementia

The age-adjusted model demonstrated no evidence of association between metformin use and dementia with HR 1.01 (95% CI 0.96 to 1.07). The minimally adjusted model, which also included sex and calendar time, estimated that metformin users experienced a lower risk of incident all-cause dementia compared with alternative GLT users with a best estimate of HR 0.83 (95% CI 0.79 to 0.88). The fully adjusted model, which included all available confounders apart from ethnicity (due to missingness), gave a similar best estimate of HR 0.87 (95% CI 0.79 to 0.94, n=146 883). Covariate missingness meant 64 513 individuals were excluded from the fully adjusted estimate. RMSE estimates were minimized in the fully adjusted model and no model reduction was required (table 2 and online supplemental table 2).

**Table 2:** Associations of metformin versus alternative GLT with dementia and MCI

Exposure	N persons	N events	HR (95% CI)	SE	RMSE	P value
**Dementia**						
Age-adjusted						
Other GLTs	32 063	1650	1 (ref)		0.162	
Mtf	179 333	4992	1.01 (0.96 to 1.07)	0.029		0.653
Minimally adjusted (adjusted by age, sex and calendar time)						
Other GLTs	32 063	1650	1 (ref)		0.046	
Mtf	179 333	4992	0.83 (0.79 to 0.88)	0.024		<0.001
Fully adjusted*						
Other GLTs	16 547	713	1 (ref)		0.044	
Mtf	130 336	3282	0.87 (0.79 to 0.94)	0.038		0.001
**MCI**						
Age-adjusted						
Other GLTs	32 063	2438	1 (ref)		0.086	
Mtf	179 333	8366	1.00 (0.95 to 1.05)	0.023		0.98
Minimally adjusted (adjusted by age, sex and calendar time)						
Other GLTs	32 063	2438	1 (ref)		0.024	
Mtf	179 333	8366	0.92 (0.87 to 0.96)	0.022		<0.001
Fully adjusted*						
Other GLTs	16 547	1105	1 (ref)		0.035	
Mtf	130 336	5814	0.92 (0.86 to 0.99)	0.032		0.017

Excludes ethnicity.

*Adjusted for age, sex, calendar time, IMD, body mass index, smoking status, alcohol excess, statin use, antihypertensive use, hypertension, asthma, COPD, liver disease, coronary heart disease, peripheral vascular disease, stroke, diabetic retinopathy, neuropathy, brain injury, depression, autoimmune disease, CKD, heart failure, skin and soft tissue infection, urinary tract infection, lower respiratory tract infection, sepsis and baseline HbA1c.

CKD, chronic kidney disease; COPD, chronic obstructive pulmonary disease; GLT, glucose-lowering therapy; HbA1c, hemoglobin A1c; IMD, Index of Multiple Deprivation; MCI, mild cognitive impairment; Mtf, merformin; RMSE, root mean-squared error.

There was evidence of interaction between GLT use and age (LRT p=0.03), but no evidence of interaction with sex (LRT p=0.37). Metformin users under 80 years experienced a lower risk of incident dementia with HR 0.78 (95% CI 0.68 to 0.89), but there was no evidence of a risk reduction in older individuals with HR 0.93 (95% CI 0.83 to 1.03) (table 3). For the primary outcome, model checking with Schoenfeld’s residuals showed borderline evidence of non-proportional hazards arising from the exposure with p=0.05 without specifying an age interaction. The log-log Kaplan-Meier survival plot demonstrated converging hazards over time (online supplemental figure 3). After specifying the age interaction as above, repeat calculation of Schoenfeld’s residuals yielded a null p=0.70.

**Table 3:** Associations of metformin versus alternative GLT with dementia and MCI stratified by age group and sex

	Exposure	N persons	N events	HR (95% CI)	P value
**Dementia**					
Age					
<80	Other GLTs	14 339	277	1 (ref)	
	Mtf	122 441	1542	0.78 (0.68 to 0.89)	<0.001
80+	Other GLTs	4725	436	1 (ref)	
	Mtf	21 401	1740	0.93 (0.83 to 1.03)	0.173
Sex					
Male	Other GLTs	5791	257	1 (ref)	
	Mtf	44 853	1256	0.87 (0.77 to 0.99)	0.03
Female	Other GLTs	3558	270	1 (base)	
	Mtf	31 499	1334	0.86 (0.76 to 0.97)	0.011
**MCI**					
Age					
<80	Other GLTs	14 339	623	1 (ref)	
	Mtf	122 441	3863	0.83 (0.76 to 0.91)	<0.001
80+	Other GLTs	4839	482	1 (ref)	
	Mtf	22 060	1951	1.05 (0.95 to 1.17)	0.35
Sex					
Male	Other GLTs	9986	587	1 (ref)	
	Mtf	75 587	2995	0.89 (0.81 to 0.98)	0.018
Female	Other GLTs	6561	518	1 (ref)	
	Mtf	54 749	2819	0.95 (0.86 to 1.05)	0.32

Interaction tests: dementia–age: p=0.03; dementia–sex: p=0.37; MCI–age: 0.0014; MCI–sex: 0.58.

GLT, glucose-lowering therapy; MCI, mild cognitive impairment; Mtf, metformin.

### Association of new users of metformin versus alternatives with MCI

The age-adjusted model showed no evidence of association between metformin use and incident MCI with HR 1.00 (95% CI 0.95 to 1.05). The minimally adjusted model indicated that metformin users experienced a lower risk of incident MCI (although more modest than for dementia) with a best estimate of HR 0.92 (95% CI 0.87 to 0.96). The fully adjusted model, conditioning on all other available confounders, gave an almost unchanged best estimate of HR 0.92 (95% CI 0.86 to 0.99, n=146 883). This MCI model was specified with the same covariates as for dementia (to ensure comparability) and RMSE estimates increased from RMSE 0.022 in the minimally adjusted model to RMSE 0.032 in the fully adjusted model (table 2 and online supplemental table 3).

For MCI, we also found evidence of interaction between GLT use and age, but no evidence of interaction with sex: we estimated a lower incident MCI risk among metformin users under 80 years with HR 0.83 (95% CI 0.76 to 0.91), but no evidence of a risk reduction in older users with HR 1.05 (95% CI 0.95 to 1.17), respectively. LRT yielded strong evidence to support an age interaction with p=0.001 for MCI (table 3).

### Sensitivity analyses

Application of serial restrictions in which dementia diagnoses made early during follow-up were excluded showed consistent HR estimates. In the post-2004 subgroup (after the advent of QOF), we found an estimated HR 0.88 (95% CI 0.80 to 0.97; n=138 444). In the post-2012 subgroup (post availability of dapagliflozin), the best estimate was HR 0.88 (95% CI 0.71 to 1.06, n=60 250), which was compatible with the null. In an analysis restricted to 96 308 individuals eligible for HES linkage, we found lower missingness for covariates (online supplemental table 4) and a best estimate that suggested a greater risk reduction among metformin users versus alternative GLT users than was seen in the main analysis (HR 0.80; 95% CI 0.71 to 0.90, n=68 614). The ‘as-treated’ analysis yielded an effect estimate HR 0.90 (95% CI 0.83 to 0.98, n=146 817) that was closer to the null (table 4). Application of serial restrictions on calendar period did not reveal a clear trend (online supplemental figure 4).

**Table 4:** Sensitivity analyses*

Exposure	N persons	N events	HR (95% CI)	P value
(1) (a) Lagged analysis excluding dementia diagnoses in first 3 months				
Other GLTs	16 042	697	1 (ref)	
Metformin	127 249	3239	0.88 (0.81 to 0.96)	0.004
(b) Excluding dementia diagnoses in first 6 months				
Other GLTs	15 498	679	1 (ref)	
Metformin	123 699	3182	0.88 (0.81 to 0.97)	0.008
(c) Excluding dementia diagnoses in first year				
Other GLTs	14 531	658	1 (ref)	
Metformin	116 211	3050	0.88 (0.80 to 0.96)	0.003
(d) Excluding dementia diagnoses in first 2 years				
Other GLTs	12 863	595	1 (ref)	
Metformin	102 177	2780	0.88 (0.81 to 0.97)	0.01
(2) Subgroup with entry post-2004				
Other GLTs	14 070	540	1 (ref)	
Metformin	124 374	2982	0.88 (0.80 to 0.97)	0.008
(3) Subgroup with entry post-2012				
Other GLTs	5261	106	1 ref)	
Metformin	54 989	681	0.88 (0.71 to 1.10)	0.26
(4) Restricted to those with HES linkage				
Other GLTs	7922	369	1 (ref)	
Metformin	60 692	1453	0.80 (0.71 to 0.90)	<0.001
(5) ’As-treated’ analysis				
Other GLTs	16 539	713	1 (ref)	
Metformin	130 278	3282	0.90 (0.83 to 0.98)	0.021

*Confounder adjustment as per fully adjusted models in table 2.

GLTs, glucose-lowering therapies; HES, Hospital Episode Statistics.

## Discussion

In this observational study of >200 000 UK adults treated for diabetes, we found evidence of a reduced risk of incident dementia and MCI among individuals initially prescribed metformin versus alternative GLT. We also found evidence that GLT interacted with age: individuals aged 40–79 years old exposed to metformin appeared to experience a lower risk of incident dementia and MCI than those on other GLTs, but this relationship was not seen in individuals aged ≥80 years. Best estimates suggest 23% lower incident dementia and 17% lower incident MCI associated with metformin use versus alternative GLT among individuals aged 40–79 years old. These findings were consistent across multiple sensitivity analyses. We found evidence of meaningful exposure misclassification in the main analysis as participants initiated additional GLT at later dates: 69% of participants in the alternative GLT group were prescribed metformin during follow-up. Although still compatible with a protective association, the ‘as-treated’ analysis yielded an effect estimate closer to the null.

Our results are consistent with established observational evidence showing that metformin use in diabetes is associated with a lower risk of neurodegenerative disease. Newby *et al*^12^ reported comparable estimates from a US retrospective cohort study using an ACNU design with HR 0.80 (95% CI 0.73 to 0.88) for all-cause dementia and HR 0.91 (95% CI 0.79 to 1.04) for MCI. Zhang *et al* reported a pooled relative risk (RR) 0.77 (95% CI 0.67 to 0.88) in a meta-analysis of 12 studies of metformin use and incident neurodegenerative diseases including Parkinson’s disease.^11^ They included high-quality population-based cohort studies, but many lacked active comparators. Other meta-analyses have, however, been conflicting: Zhang *et al* reported a protective association between metformin use and cognitive dysfunction (HR 0.90 (95% CI 0.88 to 0.92)) from 10 cohort studies (more of which had active comparators), but Ping *et al* found a null effect for the association between metformin use and neurodegenerative diseases including Alzheimer’s disease in a meta-analysis of 19 studies, which included cross-sectional studies with a higher risk of bias.^9 20^

Nevertheless, a recent Mendelian randomisation study, which by design eliminates reverse causation and most confounding, showed that genetically proxied metformin use was associated with a small reduction in Alzheimer’s disease risk.^21^ In that study, mitochondrial function and the *NDUFA2* gene were proposed as dementia protection mechanisms. Metformin has also been shown to have anti-inflammatory effects irrespective of diabetes status and it reduces accumulation of Alzheimer’s disease neuropathology in in vitro models.^22 23^

We found that metformin exposure was only associated with lower incident dementia risk for individuals under 80 years old. This is consistent with three previous US retrospective cohort studies and may be because older individuals accumulate multiple other risk factors for dementia, meaning that any potential benefit of metformin becomes increasingly negligible with age.^12 17 24^ Similarly, work on the predictive modeling of dementia risk factors has found that models with proven efficacy in younger groups are inaccurate in advanced old age.^25^ We observed that dementia rates increased in successive calendar periods (even when conditioning on age). This is consistent with sustained improvements in the routine ascertainment of dementia since 1990 in the UK, but there is still evidence of a dementia diagnosis gap.^26^

As far as we are aware, this is the largest historical cohort study investigating the association between metformin use and incident dementia to date with 211 396 participants. 98% of the UK population is GP-registered and CPRD is known to be representative of the UK general population and is comparable with census data.^13^ Findings are likely to be generalizable to the UK population with type 2 diabetes aged 40 years or more in receipt of oral GLTs. We used robust methodology for this study: the ACNU approach helps address some inherent weaknesses of pharmacoepidemiologic studies—namely confounding by indication and healthy user bias—and we used multiple sensitivity analyses to assess the contribution of other plausible sources of bias, which gave similar results.

The ACNU design intends to reduce possible confounding by indication and uses the first recorded drug prescription. This may have increased the potential for exposure misclassification, especially in a study with a long follow-up time, during which time treatment escalation or switching could occur. Around half of individuals classified as initial metformin users later received an alternate GLT, for example, due to treatment escalation, while 69% of individuals classified as alternative GLT users were later prescribed metformin. We note that the effect estimate from the ‘as-treated’ analysis was closer to the null, but was still consistent with a 10% risk reduction for incident dementia. Furthermore, we cannot be certain of participant adherence to treatment, which has been estimated to be as low as 36% in Western settings for individuals prescribed GLT.^27^ While interest is growing in the use of metformin as an ‘anti-aging’ therapy,^28^ it is unlikely that this would have contributed to exposure misclassification in our study. Although exposure misclassification is complex to evaluate, if the alternative GLT group received metformin at a later date, this may have contributed to underestimation of the effect size in the main analysis.

NICE guidelines have favored metformin as a first-line treatment since 2002^5^ and prescribing practice for diabetes has changed meaningfully—overall, 95% of individuals were prescribed metformin at least once and new participants were more likely to be prescribed metformin versus alternative GLT in successive calendar periods. Nevertheless, results restricted to later calendar entry were similar to the main analysis. In another sensitivity analysis, there was no evidence of spurious calendar effects, although low numbers of participants from 1990 to 2003 meant that this estimate was relatively underpowered.

Despite using an ACNU approach and multivariate adjustment, residual confounding, especially confounding by indication, remains a considerable risk. This could occur if the choice of GLT prescription was associated with underlying characteristics leading to dementia or MCI. Comorbidities such as renal impairment or heart failure are common among individuals with diabetes^29^ and may affect metformin prescribing.^30^ In our study, metformin users had lower baseline HbA1c measurements, were more likely to be overweight or obese and to be prescribed statins or antihypertensives than alternative GLT users. While we controlled for these variables in our analyses, it is difficult to rule out confounding by frailty or other factors that are poorly assessed in routine health records and may account for prescribing differences including therapeutic inertia among individuals with diabetes.^31^ Confounding may have biased effect estimates away from the null.

UK dementia diagnoses are most frequently made in general practice or in dedicated memory assessment services. CPRD recording of dementia has previously been evaluated and is comparable with other sources,^32^ but it is still likely to meaningfully underascertain cases. In England, the observed dementia prevalence from primary care electronic health records (EHRs) is approximately 62% of epidemiological predictions for individuals over 65 years old and is subject to considerable local variation.^33 34^ There is also a possibility that frequent users of GP services are likely to have better ascertainment of dementia, MCI and other conditions. Diagnostic codes are complex, overlapping and conditions are not necessarily coded by clinicians. Clinical diagnoses of subtypes are known to correlate poorly with postmortem neuropathological examination and individuals in advanced old age can present with overlapping pathological features.^35^ Given these concerns, we chose all-cause dementia for the primary outcome. We used comprehensive code lists to maximise ascertainment of dementia, but note that a substantial proportion of cases will not have a diagnosis in EHRs. Nevertheless, as long as outcome underascertainment is non-differential between metformin and alternative GLT users, it will not have biased our effect estimates.^36^

The substantial missingness for certain covariates, for example, ethnicity is problematic, because it is likely missing not at random, making techniques like multiple imputation unsuitable and results in a smaller population for the complete case analysis, potentially introducing selection bias. Also, missingness for covariates detailing baseline comorbidities could not be assessed as ascertainment relies solely on the presence of relevant Read codes. However, we hypothesize that individuals with diabetes are likely have more frequent GP consultations which would mitigate potential differential information bias. It is also reassuring that the fully adjusted models and minimally adjusted models gave similar estimates for both outcomes. Although the complete case analysis approach reduced the precision of estimates, it does not appear to have introduced bias. We also replicated our main findings in a HES-linked subgroup with improved record completeness.

## Conclusions

This study adds to a growing evidence base that suggests that metformin use in diabetes may be protective against dementia among individuals aged <80 years. However, although an ACNU approach can help to mitigate confounding by indication and healthy user bias, it may increase the potential for exposure misclassification, especially with long follow-up. Data missingness and likely underascertainment of dementia remain an ongoing concern when using routine health records. Future studies could use additional methods to reduce and explore confounding such as high-dimensional propensity scores and quantitative bias analysis. Triangulating evidence across other study designs that are robust to confounding including randomized controlled trials and Mendelian randomization will help to assess causality.

## Data Availability

Data may be obtained from a third party and are not publicly available. The data used for this study were obtained from the CPRD. Access to CPRD data is subject to protocol approval via CPRD’s Research Data Governance Process (see https://www.cprd.com/Data-access). Data acquisition is associated with a fee and data protection requirements. Code lists used to define health conditions in this study have been made openly available on LSHTM Data Compass: (https://datacompass.lshtm.ac.uk/id/eprint/3402/).
