# Supplementary material for: Incident dementia risk among patients with type 2 diabetes receiving metformin versus alternative oral glucose-lowering therapy: an observational cohort study using UK primary healthcare records
Source: BMJ Open Diabetes Res Care. 2024 Jan 25;12(1):e003548. doi: 10.1136/bmjdrc-2023-003548 (PMC10823924; doi:10.1136/bmjdrc-2023-003548)
Supplement: Supplementary data [file bmjdrc-2023-003548supp001.pdf]

Supplementary Material

<u>Supplementary methods</u>

Source population & cohort identification – main analysis

After identification of the initial study cohort (described in p5 in the main methods), we specified further restrictions to ensure data quality, e.g. including only CPRD 'research acceptable' records and including only records from practices that were CPRD 'up-to-standard' prior to the index date. Sense checking was also carried out to exclude individuals with illogical prescription dates e.g. recorded after death or before diabetes diagnosis. The full process of cohort identification is shown in supplementary figure 1

Source population & cohort identification – sensitivity analysis

For the post-hoc "as-treated" sensitivity analysis, LSHTM investigators extracted new data on the study population from CPRD flat files for a later CPRD build (July 2021) as the original raw data had been destroyed. Additional data on dates of all GLT prescriptions from Therapy files was extracted and merged into the existing analysis dataset. This sensitivity analysis dataset included 211,310 individuals – 86 fewer than the original study population.

Doran W, *et al. BMJ Open Diab Res Care* 2024; 12:e003548. doi: 10.1136/bmjdrc-2023-003548

BMJ Publishing Group Limited (BMJ) disclaims all liability and responsibility arising from any reliance placed on this supplemental material which has been supplied by the author(s)

Supplementary figure 1: Cohort identification

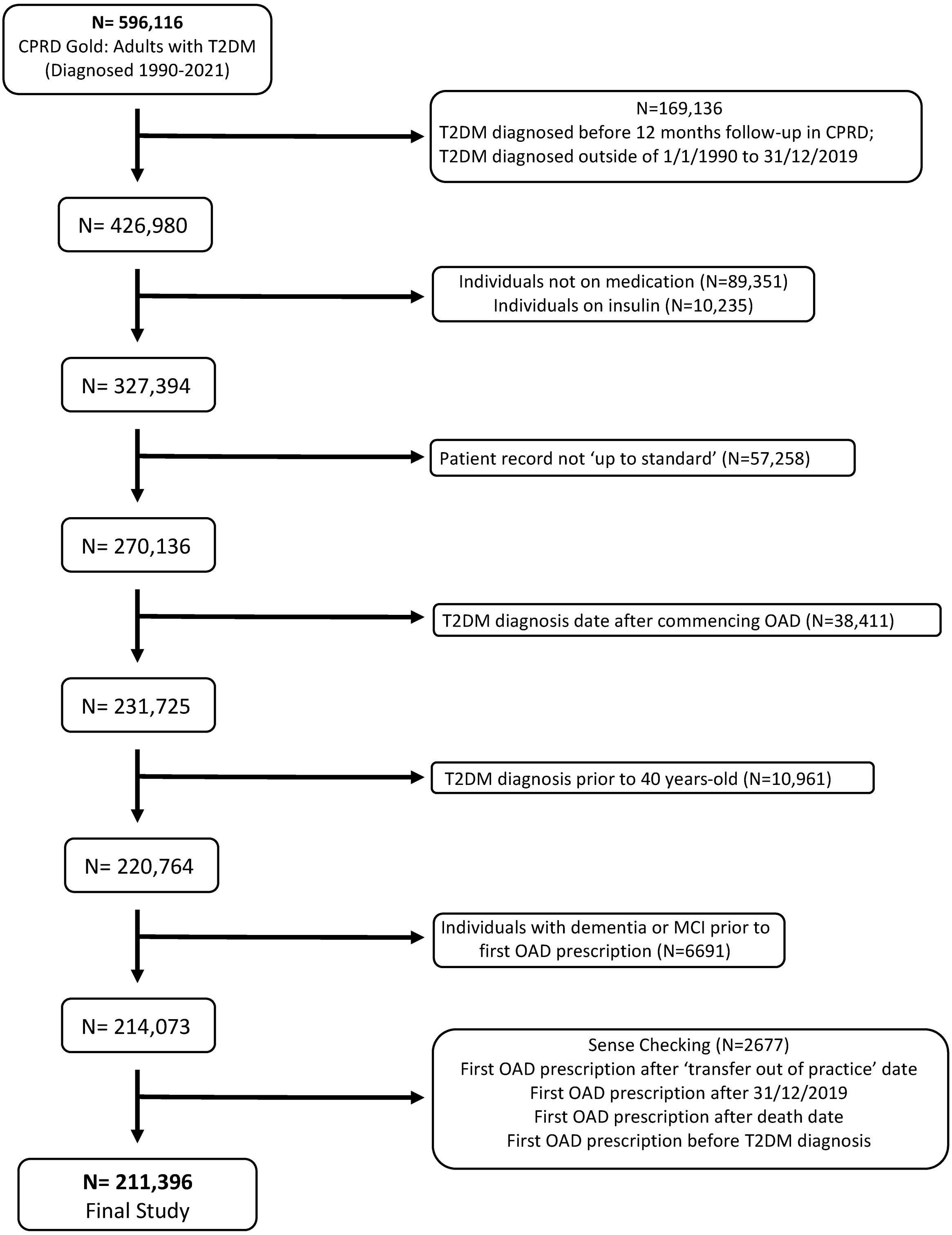

Footnote supplementary figure 1: 'Up to standard' refers to the practice 'up to standard' date, at which a practice is considered to have continuous high-quality data fit for use in research. It is derived by the data provider using an algorithm that primarily considers practice death recording and gaps in the data.

Supplementary figure 2: Directed acyclic graph

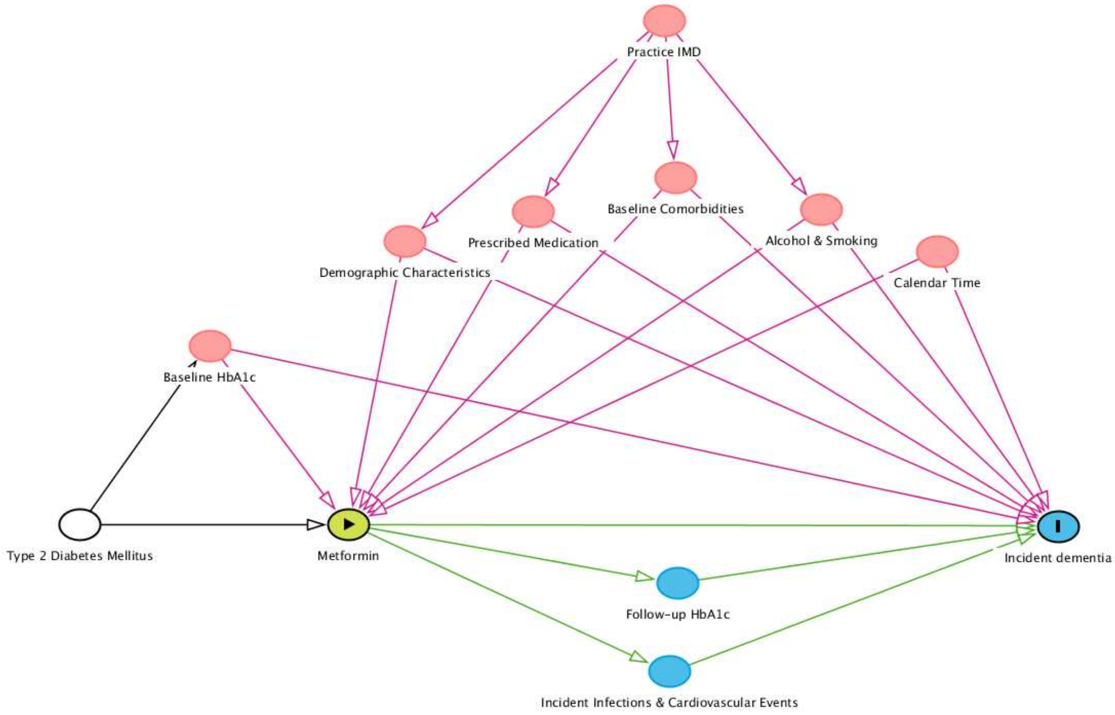

Supplementary table 1: Crude dementia & MCI rates by age band and calendar time

Variable	N Events	P-Y At Risk	Rate Per 1000 (95% CI)
Dementia			
Age bands (years)			
40-49	5	105,309	0.05 (0.02 - 0.11)
50-59	100	298,269	0.34 (0.28 - 0.41)
60-69	511	409,478	1.25 (1.14 - 1.36)
70-79	2,346	353,570	6.64 (6.37 - 6.91)
80-89	3,163	146,808	21.55 (20.81 - 22.31)
≥90	517	15,127	34.18 (31.36 - 37.25)
Calendar Time			
1990-2004	131	108,908	1.20 (1.01 - 1.43)
2005-2009	1,043	318,175	3.28 (3.09 - 3.48)
2010-2014	2,627	498,445	5.27 (5.07 - 5.48)
2015-2019	2,841	403,262	7.05 (6.79 - 7.31)
MCI			
Age bands (years)			
40-49	209	112,218	1.86 (1.63 - 2.13)
50-59	914	319,718	2.86 (2.68 - 3.05)
60-69	1,870	435,772	4.29 (4.10 - 4.49)
70-79	3,844	371,975	10.33 (10.01 - 10.67)
80-89	3,432	158,468	21.66 (20.94 - 22.39)
≥90	535	21,733	24.62 (22.62 - 26.79)
Calendar Time			
1990-2004	383	108,442	3.53 (3.20 - 3.90)
2005-2009	2,157	325,530	6.63 (6.35 - 6.91)
2010-2014	4,561	534,324	8.54 (8.29 - 8.79)
2015-2019	3,703	451,588	8.20 (7.94 - 8.47)

Supplementary table 2: Association between GLT and all-cause dementia. Covariates from fully-adjusted model.

		HR (95% CI)
**Calendar Time Band**	1990-2004	1 (ref)
	2005-2009	2.25 (1.46 - 3.45)
	2010-2014	4.08 (2.67 - 6.22)
	2015-2019	5.33 (3.5 - 8.14)
**Sex**	Male	1 (ref)
	Female	1.12 (1.04 - 1.20)
**Index of Multiple Deprivation**	1 (Poorest)	1 (ref)
	2	1.01 (0.91 - 1.13)
	3	1.05 (0.95 - 1.17)
	4	1.10 (0.99 - 1.21)
	5 (Wealthiest)	1.23 (1.11 - 1.36)
**Baseline HbA1c (mmol/mol)**	<48	1 (ref)
	48-67.9	0.95 (0.84 - 1.07)
	68-87.9	0.99 (0.87 - 1.12)
	88-107.9	0.97 (0.83 - 1.13)
	>108	0.86 (0.73 - 1.03)
**Body Mass Index (kg/m2)‡**	Underweight	1 (ref)
	Normal Weight	0.77 (0.49 - 1.20)
	Overweight	0.66 (0.42 - 1.03)
	Obesity Class I	0.58 (0.37 - 0.91)
	Obesity Class II, III	0.60 (0.38 - 0.94)
**Smoking Status**	Never	1 (ref)
	Current	1.14 (1.02 - 1.26)
	Ex	1.01 (0.94 - 1.08)
**Alcohol Misuse**		1.39 (1.05 - 1.84)
**Statin**		1.03 (0.96 - 1.11)
**Anti-Hypertensive**		1.02 (0.92 - 1.13)
**Hypertension**		0.94 (0.87 - 1.02)
**Asthma**		1.02 (0.93 - 1.11)
**COPD**		0.95 (0.86 - 1.05)
**Liver Disease**		1.19 (0.92 - 1.53)
**Coronary Heart Disease**		1.08 (1.00 - 1.17)
**Peripheral Vascular Disease**		1.31 (1.11 - 1.55)
**Stroke**		1.11 (0.74 - 1.68)
**Diabetic Retinopathy**		0.88 (0.78 - 0.99)
**Neuropathy**		1.18 (0.99 - 1.40)
**Brain Injury**		1.61 (1.21 - 2.14)
**Depression**		1.52 (1.41 - 1.63)
**Autoimmune Disease**		0.99 (0.89 - 1.09)
**Chronic Kidney Disease**		0.72 (0.47 - 1.11)

**Heart Failure**	1.14 (1.01 - 1.29)
**Skin & Soft Tissue Infection**	0.97 (0.89 - 1.05)
**Urinary Tract Infection**	1.04 (0.96 - 1.13)
**Lower Respiratory Tract Infection**	0.99 (0.92 - 1.06)
**Sepsis**	1.24 (0.91 - 1.69)

Supplementary table 3: Association between GLT and MCI. Covariates from fully-adjusted model.

		HR (95% CI)
**Calendar Time Band**	1990-2004	1 (ref)
	2005-2009	1.55 (1.25 - 1.94)
	2010-2014	1.98 (1.60 - 2.46)
	2015-2019	1.82 (1.46 - 2.26)
**Sex**	Male	1 (ref)
	Female	1.00 (0.95 - 1.06)
**Index of Multiple Deprivation**	1 (Poorest)	1 (ref)
	2	1.02 (0.94 - 1.11)
	3	1.02 (0.95 - 1.11)
	4	1.02 (0.95 - 1.11)
	5 (Wealthiest)	1.13 (1.04 - 1.21)
**Baseline HbA1c (mmol/mol)**	<48	1 (ref)
	48-67.9	0.95 (0.87 - 1.03)
	68-87.9	0.94 (0.85 - 1.03)
	88-107.9	0.85 (0.75 - 0.95)
	>108	0.91 (0.80 - 1.03)
**Body Mass Index (kg/m2)‡**	Underweight	1 (ref)
	Normal Weight	0.71 (0.50 - 1.02)
	Overweight	0.61 (0.43 - 0.87)
	Obesity Class I	0.61 (0.43 - 0.87)
	Obesity Class II, III	0.62 (0.43 - 0.89)
**Smoking Status**	Never	1 (ref)
	Current	1.08 (1.00 - 1.16)
	Ex	1.03 (0.98 - 1.09)
**Alcohol Misuse**		1.14 (0.93 - 1.4)
**Statin**		1.04 (0.98 - 1.10)
**Anti-Hypertensive**		1.03 (0.95 - 1.10)
**Hypertension**		0.93 (0.87 - 0.98)
**Asthma**		1.09 (1.02 - 1.16)
**COPD**		1.00 (0.93 - 1.07)
**Liver Disease**		1.16 (0.97 - 1.38)
**Coronary Heart Disease**		1.11 (1.04 - 1.17)
**Peripheral Vascular Disease**		1.29 (1.13 - 1.47)
**Stroke**		1.20 (0.87 - 1.64)
**Diabetic Retinopathy**		1.03 (0.94 - 1.12)
**Neuropathy**		1.16 (1.01 - 1.33)
**Brain Injury**		1.28 (1.03 - 1.61)
**Depression**		1.62 (1.54 - 1.71)
**Autoimmune Disease**		1.16 (1.08 - 1.25)
**Chronic Kidney Disease**		1.19 (0.89 - 1.59)

**Heart Failure**	1.21 (1.12 - 1.29)
**Skin & Soft Tissue Infection**	1.11 (1.05 - 1.18)
**Urinary Tract Infection**	1.16 (1.09 - 1.23)
**Lower Respiratory Tract Infection**	1.17 (1.11 - 1.23)
**Sepsis**	1.09 (0.86 - 1.39)

Supplementary table 4: Descriptive statistics for HES-linked cohort (n=96,308)

Variable (n missing)		Overall N (%$_n$)†	Metformin N (%$_n$)	Other GLT N (%$_n$)
**Totals**		96,308 (-)	81,200 (84.3)	15,108 (15.7)
**Age at Entry**	Median (IQR)	61 (53 - 70)	61 (52 - 69)	64 (55 - 73)
**Sex**	Male	54,900 (57.0)	45,932 (56.6)	8,968 (59.4)
	Female	41,408 (43.0)	35,268 (43.4)	6,140 (40.6)
**Ethnicity**	White	48,205 (88.5)	41,510 (88.4)	6,695 (89.0)
	South Asian	3,561 (6.5)	3,123 (6.7)	438 (5.8)
	Black	1,643 (3.0)	1,390 (3.0)	253 (3.4)
	Mixed or Other	1,054 (1.9)	916 (2.0)	138 (1.8)
	Missing	41,845 (43.4*)	34,261 (42.2*)	7,584 (50.2*)
**Index of Multiple Deprivation**	1 (Poorest)	18,540 (19.3)	15,685 (19.3)	2,855 (18.9)
	2	20,068 (20.8)	16,738 (20.6)	3,330 (22.0)
	3	20,801 (21.6)	17,510 (21.6)	3,291 (21.8)
	4	19,181 (19.9)	16,332 (20.1)	2,849 (18.9)
	5 (Wealthiest)	17,718 (18.4)	14,935 (18.4)	2,783 (18.4)
**Baseline HbA1c (mmol/mol)**	<48	7,353 (9.0)	6,405 (8.9)	948 (9.3)
	48-67.9	40,532 (49.4)	36,801 (51.2)	3,731 (36.4)
	68-87.9	18,739 (22.8)	16,221 (22.6)	2,518 (24.6)
	88-107.9	9,310 (11.3)	7,751 (10.8)	1,559 (15.2)
	>108	6,130 (7.5)	4,645 (6.5)	1,485 (14.5)
	Missing	14,244 (14.8*)	9,377 (11.5*)	4,867 (32.2*)
**Body Mass Index (kg/m^2)‡**	Underweight	277 (0.3)	121 (0.2)	156 (1.2)
	Normal Weight	9,712 (11.3)	6,290 (8.5)	3,422 (27.4)
	Overweight	28,372 (32.9)	23,441 (31.8)	4,931 (39.5)
	Obesity Class I	26,188 (30.4)	23,664 (32.1)	2,524 (20.2)
	Obesity Class II, III	21,608 (25.1)	20,148 (27.4)	1,460 (11.7)
	Missing	10,151 (10.5*)	7,536 (9.3*)	2,615 (17.3*)
**Smoking Status**	Non	36,710 (44.6)	31,714 (44.5)	4,996 (45.2)
	Current	14,582 (17.7)	12,515 (17.6)	2,067 (18.7)
	Ex	30,988 (37.7)	26,991 (37.9)	3,997 (36.1)
	Missing	14,028 (14.6*)	9,980 (12.3*)	4,048 (26.8*)
**Alcohol Misuse**		947 (1.0)	766 (0.9)	181 (1.2)
**Statin**		58,926 (61.2)	52,137 (64.2)	67,89 (44.9)
**Anti-Hypertensive**		65,099 (67.6)	55,426 (68.3)	9,673 (64)
**Hypertension**		48,843 (50.7)	41,898 (51.6)	6,945 (46)
**Asthma**		15,416 (16.0)	13,315 (16.4)	2,101 (13.9)
**COPD**		11,267 (11.7)	9,325 (11.5)	1,942 (12.9)
**Liver Disease**		2,078 (2.2)	1,795 (2.2)	283 (1.9)
**Coronary Heart Disease**		14,808 (15.4)	12,009 (14.8)	2,799 (18.5)
**Peripheral Vascular Disease**		1,798 (1.9)	1,459 (1.8)	339 (2.2)
**Stroke**		376 (0.4)	309 (0.4)	67 (0.4)
**Diabetic Retinopathy**		5,133 (5.3)	4,470 (5.5)	663 (4.4)
**Neuropathy**		2,084 (2.2)	1,756 (2.2)	328 (2.2)

**Brain Injury**	740 (0.8)	652 (0.8)	88 (0.6)
**Depression**	24,938 (25.9)	21,666 (26.7)	3,272 (21.7)
**Autoimmune Disease**	7,484 (7.8)	5,960 (7.3)	1,524 (10.1)
**Chronic Kidney Disease**	495 (0.5)	219 (0.3)	276 (1.8)
**Heart Failure**	3,716 (3.9)	2,735 (3.4)	981 (6.5)
**Skin & Soft Tissue Infection**	21,308 (22.1)	18,442 (22.7)	2,866 (19.0)
**Urinary Tract Infection**	18,504 (19.2)	15,709 (19.3)	2,795 (18.5)
**Lower Respiratory Tract Infection**	40,479 (42.0)	34,618 (42.6)	5,861 (38.8)
**Sepsis**	840 (0.9)	670 (0.8)	170 (1.1)

† $\%_n$: % of non-missing * % of total
‡ BMI Category Definitions (Kg/m2): Underweight <18.5, Normal Weight 18.5-24.9, Overweight 25-29.9, Obesity Class I 30-34.9, Obesity Class II & III ≥ 35

Doran W, *et al. BMJ Open Diab Res Care* 2024; 12:e003548. doi: 10.1136/bmjdrc-2023-003548

Supplementary figure 3: Log-Log Kaplan Meier Survival Plot

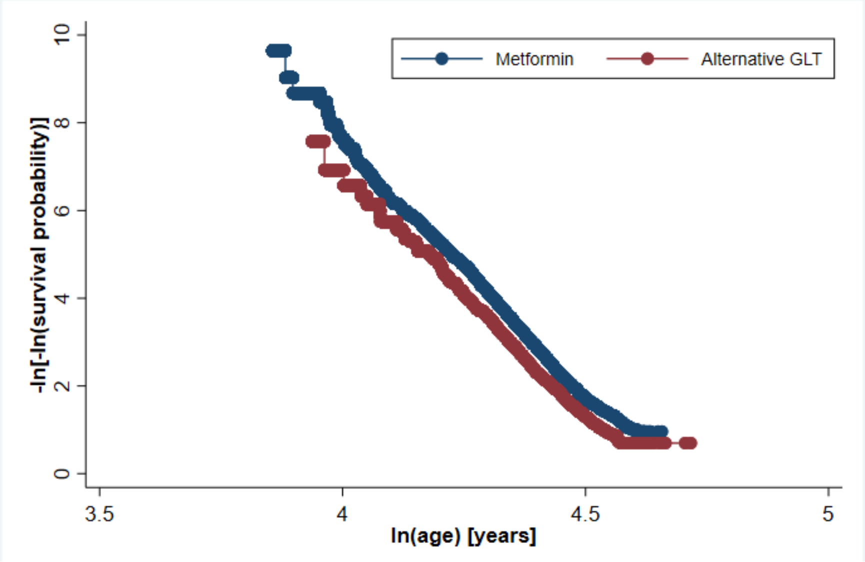

Supplementary figure 4: Serial restrictions on calendar period

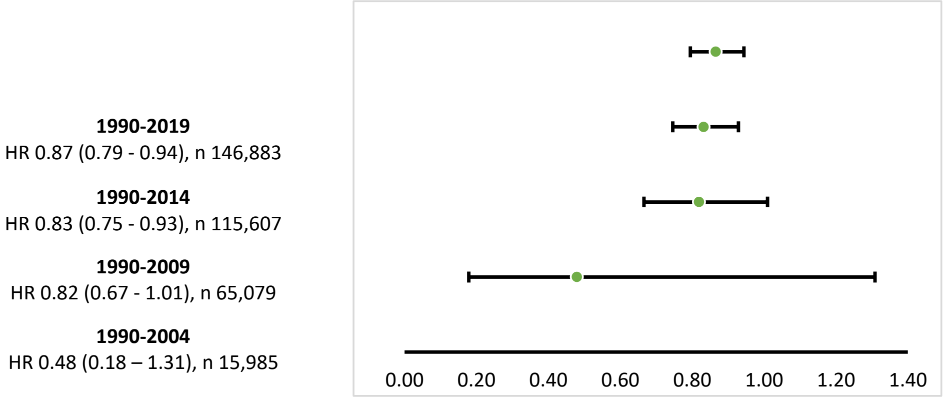

Doran W, *et al. BMJ Open Diab Res Care* 2024; 12:e003548. doi: 10.1136/bmjdrc-2023-003548
